# Oral Microbiota Dysbiosis in Firefighters and the Potential Contributing Environmental and Lifestyle Factors Based on a Case-Control Study

**DOI:** 10.3390/microorganisms13051154

**Published:** 2025-05-18

**Authors:** Sukanta S. Bhattacharya, Brijesh Yadav, Roman Jandarov, William A. Jetter, Jagjit S. Yadav

**Affiliations:** 1 Pulmonary Pathogenesis and Immunotoxicology Laboratory, Department of Environmental and Public Health Sciences, College of Medicine, University of Cincinnati, Cincinnati, OH 45267, USA; bhattacharya.s.s@gmail.com (S.S.B.); brijeshsgpgims@gmail.com (B.Y.); 2 Department of Biostatistics, Health Informatics & Data Sciences, College of Medicine, University of Cincinnati, Cincinnati, OH 45267, USA; jandarrn@ucmail.uc.edu; 3 The Monroe Fire Department, Cincinnati Metropolitan Area, Monroe, OH 45050, USA; jetterbj@cinci.rr.com; 4 Fire Science, College of Engineering and Applied Science, University of Cincinnati, Cincinnati, OH 45221, USA

**Keywords:** firefighters, oral microbiome, dysbiosis, occupational exposure, cancer, PAHs

## Abstract

Epidemiological studies show firefighters have increased risks of cancer, diabetes, and cardiovascular disease. To explore links between occupational/environmental exposures and dysbiosis-associated health risks, this case-control study compared oral microbiota of age-matched firefighters (n = 13) and non-firefighters (n = 13) using next-generation sequencing. Firefighters exhibited significantly reduced overall microbial diversity (*p* ≤ 0.05) and compositional shifts. Firmicutes increased from 53.5% to 68.5%, and Bacteroidetes from 9.5% to 14.1%, while Proteobacteria decreased from 24.6% to 8.3%, and Fusobacteria from 3.3% to 1.1%. This resulted in a higher Firmicutes to Bacteroidetes ratio (5.63 vs. 4.89 in controls), indicating a pro-inflammatory oral microenvironment. At the family level, Streptococcaceae (45.1% to 60.3%) and Prevotellaceae (6.2% to 10.0%) increased, whereas Neisseriaceae (17.7% to 4.9%) and Fusobacteriaceae (2.1% to 0.8%) decreased. The genus *Streptococcus* dominated firefighters’ microbiota, rising from 45.1% to 60.3%. Diversity indices confirmed reduced microbial evenness and richness in firefighters. Metadata analysis linked frequent fire exposures to perturbations in Comamonadaceae and Carnobacteriaceae (*p* ≤ 0.05). Barbecue consumption, a source of polycyclic aromatic hydrocarbons, correlated with elevated Spirochaetaceae and Peptostreptococcaceae. This first report on oral dysbiosis in firefighters reveals significant alterations in microbiota abundance, diversity, and evenness, implying potential health risks for this group.

## 1. Introduction

Firefighters are regularly exposed to heat and smoke, involving inhalation of particulate matter (PM) and chemicals [1]. Fire-associated smoke contains PM sizes of concern, as well as several proinflammatory and carcinogenic chemicals, such as polycyclic aromatic hydrocarbons (PAHs), benzene, formaldehyde, chlorophenols, dioxins, ethylene oxide, diesel fumes, arsenic, and asbestos, among others [2]. These exposures have been linked to upper respiratory tract (including the nasal, oropharyngeal, and laryngeal regions) and lower respiratory tract conditions/symptoms as well as systemic pathologies [3]. Case-control etiological studies have linked occupational carcinogen and toxicant exposures to the induction of various carcinomas, including head and neck, lung, prostate, colon, and bladder cancers, and other systemic diseases [4,5,6,7,8]. For instance, occupational groups such as firefighters, agricultural workers, construction workers, and roofers have been identified to be at a higher risk of Head and Neck Squamous Cell Carcinoma (HNSCC) [9]. Earlier studies carried out at this Department at the University of Cincinnati on firefighters have revealed high concentrations of PAHs on the firefighters’ bodies, which potentially exposes them to the risk of cancer [10]. Besides, independent studies have revealed that firefighters have an increased risk of Type 2 diabetes, cardiovascular diseases, as well as systemic inflammatory diseases [1,10,11,12]. Collectively, these trends are indicative of the role of environmental/occupational risk factors during firefighting in the development of various diseases [4,5,13]. These disease risks have been conventionally attributed to the occupational exposure to abiotic factors, particularly smoke and associated toxicants. However, there are strong indications of the involvement of biotic factors playing role in the elevated risk of these diseases [14,15]. In this context, studies on the general human population provide increasing evidence on the role of oral microbiota in the etiology of some of these significant diseases [14,15,16]. For instance, oral microbiota has been linked to cancer [13,17] and other systemic diseases, including diabetes [14], cardiovascular disease [18], and stroke. Strong evidence exists underlying the role of oral microbiome in the onset of HNSCC, including Oral Squamous Cell Carcinoma (OSCC) [13,15,19,20]. In this context, studies have reported an association of oral hygiene and dental disease (which impact oral microbiota) with HNSCC [10,21,22,23]. Independent studies have shown that tobacco smoke alters oral microbial flora [24,25]. However, the interaction between fire-associated exposures (such as smoke, heat, and particulates) and the oral microbiome is yet to be understood. Hence, in this study, we evaluated the oral microbiome composition and diversity in firefighters and control subjects to investigate the changes in microbiome constituents associated with the occupational (firefighting) and related lifestyle risk factors. To our knowledge, this is the first report on the characterization of oral microbiome perturbations in firefighters and may form the basis of larger studies to provide a better understanding of the microbial etiology of health risks in firefighters.

## 2. Materials and Methods

### 2.1. Study Population

**Figure 1:**
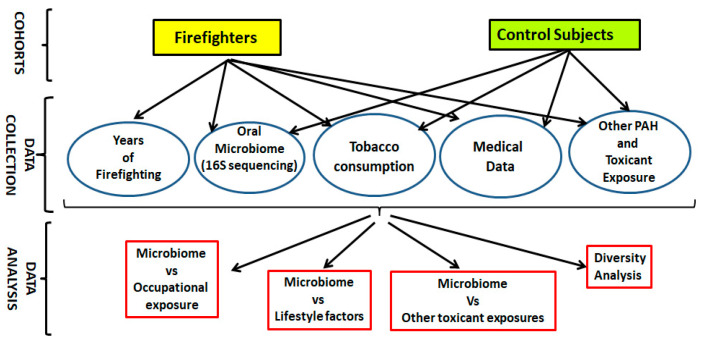
Study workflow used to evaluate shift in oral microbiome in firefighters.

Thirteen ethnicity- and age-matched firefighters (who were actively engaged in firefighting) and 13 control subjects (who were non-firefighters and non-smokers) from the Greater Cincinnati area were recruited, following our IRB-approved protocol. Only male firefighters with more than three years in firefighting service were enrolled in the present study. The study subjects were in the younger age range, with the majority being below 35 years of age. Subjects with a history of cancer, dental diseases, enlarged prostate, and urinary bladder infection, as well as those who had used any antibiotics within the preceding two weeks, were excluded from the current study. The enrolled subjects were asked to provide their oral rinse generated by swishing the mouth/oral cavity for 30 s using 30 mL of sterile physiological saline (0.9% *w*/*v* normal saline) [26]. The oral rinse samples were transferred to the host laboratory on ice. The study was conducted per the scheme outlined in Figure 1 and as elaborated in the subsequent sections.

### 2.2. Metadata Collection

Each study subject was asked to complete an IRB-approved study questionnaire that included specific questions meant to assess environmental/occupational and non-occupational factors, such as health and lifestyle factors, that may potentially impact the oral microbiome. For instance, the occupational factors were assessed by asking participants to answer questions on the length and magnitude of their firefighting experience, among others. Lifestyle questions included those meant to assess other ways of intake of PAHs, such as via smoking and other methods of tobacco use, and the consumption of barbequed foods; the dietary PAH intake was estimated as milligrams/day using the per serving values described in previous reports [27]. Other questions included prior medications, including antibiotics, practices related to oral hygiene, family history of cancer, and any other health issues that may potentially affect oral microbiota.

### 2.3. Processing of Oral Microbiota Samples and Isolation of Microbial DNA

The oral rinse samples were processed for isolation of total microbial DNA, using Qiagen’s DNeasy Blood and Tissue kit (Qiagen Inc., Germantown, MD, USA), following the manufacturer’s protocol with modifications. Briefly, a 2 mL aliquot of oral rinse was centrifuged at ≥10,000 rpm for 10 min. The resulting pellet containing microorganisms was resuspended and lysed by treating with the modified lysis buffer (20 mM Tris-HCl, pH 8.0, 2 mM Sodium EDTA, 1.2% Triton X-100 supplemented immediately before use with cell wall lytic enzymes including 20 mg/mL Lysozyme and 40 IU/mL Mutanolysin) at 37 °C for 30 min in conjunction with subsequent bead beating [28]. Buffer AL of the kit was then added to the crude lysate, followed by purification of the microbial DNA using the kit’s DNeasy Mini Spin column per the manufacturer’s protocol. The extracted DNA was stored at −80 °C until further analysis.

### 2.4. Analysis of the Microbiome

The isolated microbial DNA samples were quantified using a UV-VIS spectrophotometer, the NanoDrop 2000 (Thermo Scientific, Waltham, MA, USA). The DNA samples were assessed for quality in terms of PCR amplifiability by targeting ~300 bp of the variable region-4 (V4) of the 16S rRNA gene using FastStart Taq DNA Polymerase kit (Roche Diagnostics, GmbH, Germany). The primer set (Forward primer 515F and Reverse primer 806R) used for this quality check step was the same as employed for the downstream next-generation sequencing of the library of 16S rRNA gene amplicons, except for the incorporation of flanking bar codes in the latter set, and is known to yield the maximum prokaryotic taxonomic group coverage [28,29]. The primer sequences were as follows: forward primer 515F (5′-GTG CCA GCM GCC GCG GTA A-3′) and reverse primer 806R (5′-GGA CTA CHV GGG TWT CTA AT-3′). In the downstream next-generation sequencing protocol, these two primers were tailed with Illumina flow cell-compatible sequences and two sets of 24 different 12-nucleotide molecular barcodes each, allowing up to 576 communities to be sequenced together on the same flow cell. The next-gen sequencing was done on an Illumina MiSeq platform using MiSeq V2, 500-cycle kit (Illumina, San Diego, CA, USA), following the method described in previous reports [30]. A sample sheet was prepared without demultiplexing options since the barcodes are in line with the sequence read, and the MiSeq run was set up for FASTQ generation, using only 251 bases with paired ends. The results delivered were from Illumina’s ‘MiSeq Reporter FASTQ’s only’ workflow. The run metrics stored in the InterOp folder were evaluated for runtime quality checks.

Data analysis, which included reducing PCR errors, processing improved sequences, assessing error rates, clustering into Operational Taxonomic Units (OTUs), and phylogenetic analysis, was performed using the program Mothur (v.1.43.0), following the standard operating procedure [31], alongwith the SILVA-based bacterial database. The family-level analysis of microbial diversity within a cohort (α diversity) was done using the Shannon diversity and evenness indices. All the calculations were done using Microsoft Excel. The Shannon diversity index (H) was calculated as follows: H =$-\sum_{x=0}^{s}\;p_{i}.{lnp}_{i},$ where H is the Shannon diversity index, and $p_{i}$ is the proportion of the family relative to the total families. Shannon’s evenness index or Shannon’s equitability was calculated as: $E_{H}=\frac{H}{H_{max}},$ where *E_H_* is the Shannon’s equitability index, *H* is the Shannon’s index, and *H_max_* is calculated as log_n_*S*, where *S* is the total number of families present in the sample. For estimating the microbial diversity between the cohorts (β diversity), we used two different indices, namely the Bray-Curtis dissimilarity index (or index of dissimilarity) and the Sorensen index of dissimilarity. The Bray-Curtis index of dissimilarity was calculated as follows: $BC=\frac{\sum_{i=1}^{n}\left|X_{ij}-X_{ik}\right|}{\sum_{i=1}^{n}\left|X_{ij}+X_{ik}\right|}$ where BC is the Bray-Curtis index of dissimilarity, *X_ij_* and *X_ik_* are the number of individuals in cohorts j (firefighters) and k (control subjects), respectively, and n is the total number of families in the sample. Sorensen’s index of dissimilarity was calculated as follows: $\beta =\frac{b+c}{2a+b+c}$ where a is the number of families shared between two cohorts, and b and c are the number of families unique to each sample. To estimate the species richness, the Chao1 index was calculated as *S_chao_*_1_ *= S_obs_ + F*_1_^2^/2*F*_2_ where *S_chao_*_1_ is the Chao1 index, *S_obs_* is the observed number of families, *F*_1_ is the number of singleton families, and *F*_2_ is the number of doubletons.

### 2.5. Statistical Analyses

Power calculations for the study cohorts were performed considering the means of Shannon’s diversity indices and Chao1 diversity indices using the online tool: https://clincalc.com/stats/samplesize.aspx (accessed on 18 March 2025). Firefighters group, considered as the first study group, was compared to the second study group (control group) targeting 80% power, 95% confidence interval, and a 5% of margin of error. The n values obtained from the above power calculations are 11 and 5 subjects, respectively, for each group. A post-hoc analysis using the Chao1 index and a sample size of 13 (actually used in this study) for each group gave a power of 84.1%, which is deemed acceptable in environmental exposure/toxicological studies.

Microbiome analysis outputs obtained in terms of total bacterial count and individual bacterial family’s count and its relative proportion (percent of the total count) for the firefighter group and the control group were compared by the Wilcoxon sign rank-sum test using R package software (Version 0.3). Further analysis was performed for individual bacterial family’s count and proportion within the firefighter group to compare potential influencing factors (such as years of service in firefighting, number of firefighting events participated before sampling, tobacco use, smoking, dietary PAHs-intake in the last 8 h before sampling), using the Wilcoxon signed-rank test. A *p*-value ≤ 0.05 was considered to represent statistical significance.

## 3. Results

### 3.1. Population Characteristics

The study subjects were recruited from the same geographic location (in and around the Tristate area, Greater Cincinnati, OH, USA) and were of the same gender (male), age range, and ethnic background (Caucasian). However, their exposure history to environmental and other lifestyle factors varied. Briefly, the mean age of the firefighters was 28.37 + 3.31 years, while that of the control group was 32.76 + 1.37. Unlike the control subjects, who were non-firefighters, ~40% of the firefighters had more than five years of firefighting experience and had participated in more than 20 firefighting operations, each lasting more than one hour. About 31% had participated in fires lasting more than an hour within the last 30 days. In terms of lifestyle factors, while none of the control subjects used tobacco in any form, about 46% and 23% of the firefighters smoked tobacco and used smokeless tobacco, respectively. About 38% of the firefighters were exposed to barbecued food consumption within 8 h of sampling. These and the other details were taken into consideration while comparing the firefighters and the control subjects. The exclusion criteria, as described in Section 2.1, were common between the controls and the firefighters.

### 3.2. Alterations in the Oral Microbiota of Firefighters

**Figure 2:**
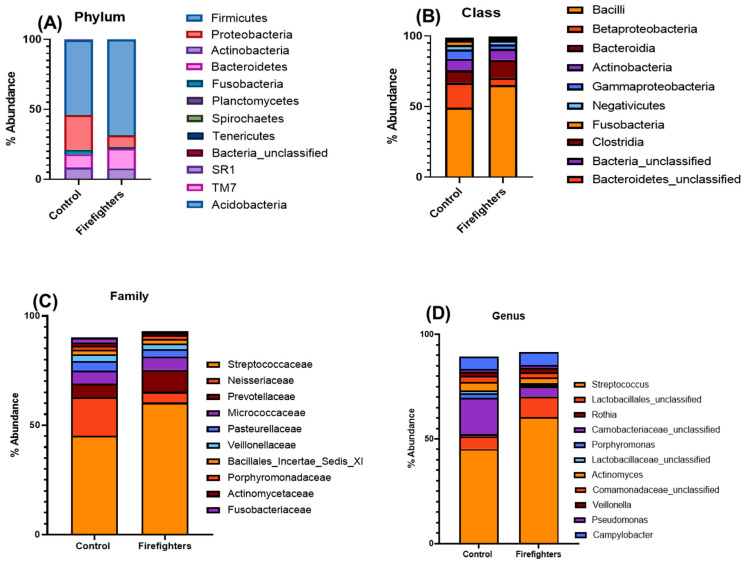
Oral dysbiosis in firefighter group as compared to the control group, depicted in terms of relative abundance (%) of major Phyla (**A**), Classes (**B**), Families (**C**), and Genera (**D**).

Oral microbiome sequences from the study subject groups were analyzed at the bacterial phylum, class, family, and genus/species levels (Figure 2A–D). The phyla showed a significant increase (*p* ≤ 0.05) in Firmicutes (53.5% to 68.5%) and Bacteroidetes (9.5% to 14.0%), and a significant decrease in Proteobacteria (24.6% to 8.3%) and Fusobacteria (3.3% to 1.1%) in firefighter group as compared to the control group (Figure 2A). There was an increase in overall Firmicutes:Bacteroidetes ratio in the firefighter group (5.63) as compared to the control group (4.89).

Bacterial family-level comparison showed some significant microbiome alterations in the firefighter group in terms of both total count and proportion of individual bacterial families detected (Table 1). A total of 26 bacterial families were impacted in this group due to firefighting (threshold value for fold change ≥ 2-fold) (Supplementary Figure S1). A significant increase (*p* ≤ 0.05) in the proportion of the bacterial family Streptococcaceae (60.28% of the total bacterial population) was observed in firefighters as compared to control subjects (45.10%) (Figure 2C). The firefighter group also demonstrated a considerable increase in Prevotellaceae (to 10.02%) and Micrococcaceae (to 6.25%) compared to the control group (6.22% and 5.85%, respectively). Further comparison with the control group showed a decrease in the firefighter group in terms of proportions of Neisseriaceae (17.7% to 5.0%), Fusobacteriaceae (2.1% to 0.8%), Pasteurellaceae (4.43% to 3.21%), and Veillonellaceae (3.22% to 2.69%). Additionally, a decreasing trend in the proportions of the bacterial families Porphyromonadaceae and Actinomycetaceae was observed in the firefighter group (Figure 2C).

### 3.3. Genus/Species-Level Alterations in the Oral Microbiota of Firefighters

Further analysis at the genus and species levels demonstrated significant qualitative and quantitative shifts. *Streptococcus* was the most abundant bacterial genus in the firefighter group (60.27%). In all, 67 genera were found to be the top impacted (using ≥2-fold change as threshold) ones in firefighters as compared to the control group, implying their association with the firefighting occupation (Table 2). Of these, 31 genera showed a substantive but variable decrease in abundance in firefighters (−2 to −26,370-fold), including *Pseudomonas* (Pseudomonadaceae family), *Arcobacter* and *Campylobacter* (Campylobacteraceae family), *Shewanella* (Shewanellaceae family), *Neisseria*, *Eikenella* sp. and *Bergeriella* (Niesseriaceae family), and *Acetoanaerobium* (Peptostreptococcaceae family). On the other hand, certain genera showed a substantiative fold-increase (2- to 35.87-fold) in the firefighter group, including *Conchiformibius* (Niesseriaceae family) and *Moraxella* (Moraxellaceae family). Notably, some of the genera were differentially detected only in the firefighter group, namely *Uruburuella*, *Oceanotoga*, *Oceanotoga*, *Acetoanaerobium*, *Rhodococcus*, *Nitrincola*, *Burkholderia*, *Ralstonia*. Similarly, a few bacterial genera that were differentially nondetectable in the firefighter group were *Mycobacterium*, *Brevundimonas*, *Yersinia*, and *Sulfurospirillum* (Table 2).

### 3.4. Microbial Diversity in the Oral Microbiome in Firefighters

**Figure 3:**
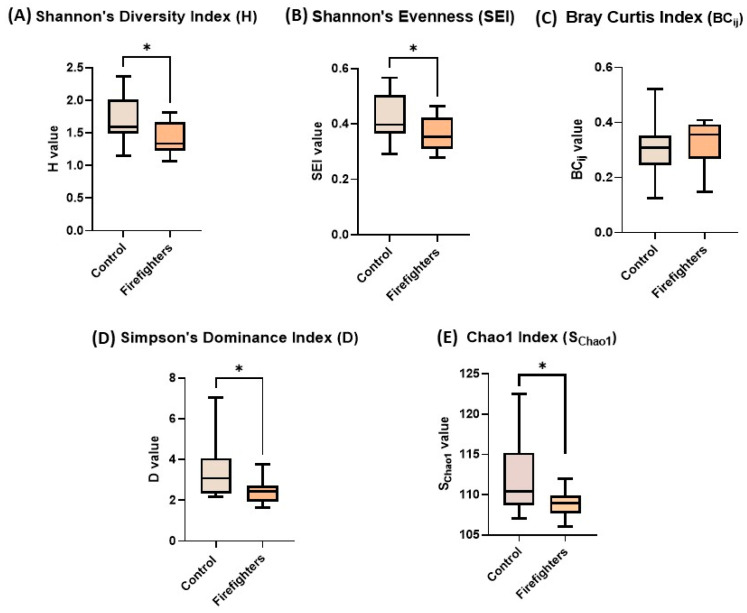
Oral microbiota alpha- and beta-diversity changes in firefighters group as compared to the control group, depicted in terms of the Shannon diversity index (**A**), Shannon evenness index (**B**), Bray-Curtis index (**C**), Simpson dominance index (**D**), and Chao1 index (**E**). * Depicts statistical significance (*p* ≤ 0.05).

To gain further insights into the microbiome dysbiosis, the microbial diversity and community structure were analyzed across multiple metrics (Figure 3). The Shannon diversity indices at the genus/species level demonstrated significantly reduced α-diversity in firefighters (1.424) compared to controls (1.71; Figure 3A). Similarly, species evenness decreased markedly in the firefighter cohort (0.3643) vs. control (0.4241) (Figure 3B), consistent with observed reduction in abundance for specific taxa (Table 2). Chao1 richness (Figure 3E) estimates at the family level revealed significantly (*p* < 0.01) diminished microbial richness in firefighters (108.8 ± 1.85) relative to controls (112.3 ± 4.48), highlighting depletion of rare taxa. β-diversity analysis did not show marked compositional divergence between groups, with insignificant differences in Bray-Curtis dissimilarity (Figure 3C) and complementary Sørensen analysis (dissimilarity index: 0.214) indicating lack of substantial family-level differences. However, Simpson’s Dominance Index (Figure 3D) values (controls: 3.47 vs. firefighters: 2.47) confirmed greater taxonomic uniqueness in controls, suggesting a loss of specialist species in the firefighter cohort. These multi-metric analyses collectively demonstrate significant restructuring of microbial communities in firefighters, characterized by reduced diversity, diminished rare taxa representation, and altered community composition compared to controls.

### 3.5. Factors Contributing to Oral Dysbiosis and Potential Occupational Health Risks in Firefighters

The results further showed that several firefighter-associated factors significantly perturbed the absolute counts and proportion of the oral microbiota. Firefighters with >5 years of firefighting service showed significant decrease in absolute count and proportions of Carnobacteriaceae and increasing trend in the GammaProteobacteria and Peptococcaceae_1 family, whereas firefighters who participated in >20 firefighting events showed significantly increased absolute number and proportion of the Comamonadaceae family. Consumption of foods containing PAHs within 8 h before sampling was associated with a significant increase in counts and proportion of bacterial families Spirochaetaceae, Lactobacillaceae, and Peptostreptococcaceae, whereas tobacco consumption during this time window significantly decreased the absolute count and proportion of Fusobacteriaceae and Comamonadaceae. Similarly, firefighters who consumed ≥3 tins/week of tobacco had decreased counts of the Spirochaetaceae family. Furthermore, firefighters using ≥1 cycle of mouthwash daily showed significantly decreased bacterial families of Comamonadaceae and Spirochaetaceae (Table 1). While ‘antibiotic use within past 2 weeks’ was one of the defined exclusion criteria for enrolling subjects in the current study, all enrolled subjects were queried for any antibiotic use within the past 30 days. While all participants satisfied the ‘no antibiotic use within 2 weeks’ criterion, only one of the participating firefighters replied in affirmative for ‘antibiotic use within last 30 days.’ To see if this impacted our overall conclusions, we compared this individual’s microbiome profile with the overall profile of the firefighter group. Notably, this individual’s profile was found to fall within the range for the group, and the differences, if any, were non-significant (*p* > 0.05). For instance, the microbial index values for this individual, namely, Chao1 index (110.17), Simpson’s index (2.44), and Shannon index (1.52), fell within the observed range for the respective indices for the group (Figure 3). In this context, it is worth noting that controlled studies using animal models have shown the resilience of oral microbiome to the use of antibiotics [32], while other studies have reported the ability of the oral microbiome to attain the baseline in a month of antibiotic treatment [33]. Overall, while the literature on time interval for reversal of microbiota after antibiotic use varies, we have considered one month as a reasonable time for reversal in the current study.

## 4. Discussion

Firefighters are known to be at an increased risk from several occupational exposure-linked diseases, and there is a continuing interest in identifying their specific etiological agents and biomarkers. Though non-biological factors have been implicated, little if anything is known about the biological factors, and there is no information available on the role of a firefighter’s own microbiome in the onset of these diseases. In this context, there is a paucity of integrative studies linking the alteration of microbiome with the length of occupational exposure and the onset of occupational diseases in high-risk professions such as firefighting. The current study focuses on oral microbiome changes in the firefighting occupational group along with the underlying contributing factors. The overall objective was to understand the nature of microbiome perturbations and their similarity to dysbiosis constituents implicated in health risks in the adult human population.

The current study showed a clear perturbation in oral microbiota constituents (oral dysbiosis) in firefighters. At the phylum level, there was a significant increase in Firmicutes:Bacteroidetes ratio and a significant decrease in Proteobacteria and Fusobacteria in firefighters. At the class/family level, an increase in the class Bacilli and the families Streptococcaceae and Prevotellaceae and a decrease in the family Fusobacteriaceae were observed. At the genus level, a significant increase in *Streptococcus* and a decrease in *Porphyromonas* were observed in firefighters. An elevated overall Firmicutes:Bacteroidetes ratio implied a pro-inflammatory oral microenvironment in the firefighter group. This may have health implications as suggested by studies on other human cohorts [34,35]. For instance, a higher abundance of oral microbiota phylum Firmicutes and its constituents, including class Bacilli, families Streptococcaceae and Prevotellaceae, and genus *Streptococcus*, have been associated with Parkinson’s disease [36]. An increase in the proportion of Streptococcaceae in the oral cavity was reported in cancer patients [37] and in those who showed greater susceptibility to respiratory tract infections [3]. Decreases in the bacterial family Neisseriaceae and its genus *Neisseria*, as observed in the current study on firefighters, were positively associated with higher depression scores [36]. Furthermore, a higher abundance of *Moraxella* spp., as observed in our firefighter cohort, has been reported to be linked with human endocarditis [38]. A decrease in Actinobacteria and an increase in Bacteroidetes abundance, as observed in the current study, have been linked to OSCC [20].

On the other hand, the control subjects showed microbiome characteristics and trends consistent with oral eubiosis in healthy adult individuals [39,40]. For instance, predominant families in healthy individuals include Streptococcaceae (with *Streptococcus* comprising approximately 22–54% of total abundance), Neisseriaceae (*Neisseria*, ~14%), Pasteurellaceae (*Haemophilus*, ~14%), Veillonellaceae (*Veillonella*, high prevalence), Micrococcaceae subfamily Rothiaceae (*Rothia*), and Actinomycetaceae (*Actinomyces*). Collectively, these families account for over 80% of the oral microbial community in healthy adults. At the phylum level, healthy oral microbiomes primarily show the following order of abundance: Firmicutes > Proteobacteria > Bacteroidetes > Fusobacteria/Actinobacteria [39]. While subtle differences exist in the abundance values of certain OTUs reported in the literature and our current study, this may be attributed to differences in microbiome sampling, DNA extraction procedures, and analysis. While most of the reports have used saliva or an oral swab as their sample, we preferred to use an oral rinse, which captures the oral bacterial diversity more holistically than the other sampling techniques.

Human epidemiological studies have specifically reported associations between altered oral microbiome (oral dysbiosis) and incidences of cancer and other systemic diseases [13,36,41]. An altered oral microbiome may occur due to the alteration in physiological conditions in the buccal cavity, such as in response to stress, lifestyle factors (tobacco smoke, alcohol), and/or health conditions such as periodontal diseases [40]. Firefighters are one of the professional groups who are at risk of occupational exposure to smoke, carrying several recognized, as well as probable, carcinogens and multiple stress factors, such as heat exposure and stressful working hours. Particularly, in the firefighter group sampled in the present investigation, the personnel were on duty for 24 h followed by a 48 h off-duty period. This contrasts with the regular service personnel in other professions who have an 8-to-10-h duty period. These work conditions for firefighters may differentially alter the oral microbiome, which in turn may exert an effect on the metabolism/physiology, predisposing firefighters to several associated health risks and ensuing disease conditions [42]. This implies that the oral microbiome perturbations in firefighters in this study could be due in part to the associated occupational stress, besides their exposure to fire smoke and other fire-related factors.

The role of bacteria in causing/promoting cancer has been well established, such as *Helicobacter pylori* in gastric cancer [43] and other bacterial species in cancers of the gallbladder, colon, lung, and prostate [44]. A shift in the oral microbiota is also reported in individuals with oral/head and neck cancer [10,15,17] and other health conditions. Nevertheless, independent reports also exist on the alteration of the oral microbiome in healthy individuals who were exposed to tobacco smoke PAHs or alcohol [24,25,45,46]. In the current study, we observed a shift in oral microbiome in terms of proportions of several bacterial families in firefighters when compared with control subjects (Table 1). For instance, firefighters who had >5 years firefighting experience and who underwent >20 firefighting events since they joined firefighting job showed a significantly increased (*p* ≤ 0.05) proportion of Peptostreptococcaceae. Likewise, within the firefighter group, those who ate grilled barbecue food (a recognized source of PAHs) within 8 h before the sampling had a considerably elevated count and proportion for Spirochaetaceae, Lactobacillaceae, and Peptostreptococcaceae. A similar trend was observed for Eubacteriaceae in firefighters who used tobacco 8 h before the sampling, although Fusobacteriaceae and Comamonadaceae counts were significantly lower in firefighters. These findings revealed the nature of oral microbiome dysbiosis in firefighters and their potential for predisposition to health risks in the long-term. For instance, an altered oral microbiota is suspected to play a role in the induction of head and neck cancers (HNSCC, OSCC) [23]. Initial studies in this direction have indicated significant differences in oral microbiome between smokers, HNSCC patients, and healthy individuals [47,48], although specific risk factors causing such differences are yet to be understood. In this context, human microbiomes have been shown to contribute to xenobiotic/carcinogen biotransformation or bioactivation either directly [49] or via modulation of host cells [50]. A similar scenario, conceptualizing the bioactivation of carcinogenic chemicals such as PAHs by an altered oral microbiota, may apply to firefighters. Oral microbial dysbiosis may also, in part, play a modifier role in the host cell-toxicant/carcinogen (e.g., smoke-associated PAHs) interaction that may, in turn, be responsible for the onset and/or progression of cancers in firefighters. Such interactions may also have a role to play in other systemic diseases such as cardiovascular, diabetes, systemic, and pulmonary inflammation diseases. Collectively, these facts strongly support the argument that the occupational risks of cancer and other diseases in the firefighting profession may be a consequence of an altered oral microbiome diversity (oral dysbiosis) due to hazardous exposures. In this context, the observed overall reduced oral microbiome diversity in firefighters (as indicated by decreased values of the Shannon diversity index, Simpson dominance index, and Chao1 index) may be due to the selection pressure on the oral microbial species in the firefighters, presumably because of their occupational exposure to the fire smoke, dust, and/or associated chemicals during firefighting operations. Some of these differentially impacted species could be further validated as potential biomarkers for assessing exposure risks in firefighters or potentially other cohorts with similar occupational exposures.

Furthermore, a few genera, including *Uruburuella*, *Oceanotoga*, *Enterococcus*, *Acetoanaerobium*, *Rhodococcus*, *Burkholderia*, and *Ralstonia*, were uniquely found in firefighter samples. In terms of their known clinical relevance, studies have associated *Enterococcus* with endocarditis and vancomycin resistance [51], and *Rhodococcus* spp. with pneumonia in immunocompromised individuals [52]. *Rhodococcus* spp. and *Burkholderia* spp. are also known for their ability for biotransformation of aromatic hydrocarbons (monoaromatic, polycyclic aromatic, and heterocyclic) [53,54], to which firefighters are routinely exposed from fire smoke emanating during firefighting service.

*Limitations and strengths of the study*: To our knowledge, the current study is the first to have analyzed the oral microbiome of firefighters and factors associated with perturbation of the oral microbiome. While the study compared cases and controls of the same age range, ethnicity, and geographic location, it is based on a limited sample size. While the results showed significant perturbation in the oral microbiome of the firefighter group, the comparison between the subgroups within this group had limited power for statistical comparison. Nonetheless, some of the subgroup comparisons showed statistically significant trends. Considering that microbiome analysis was based on the 16S rRNA V4 region, the species level resolution was not achieved. Further studies on a larger cohort of firefighters using shotgun sequencing will help validate the observed oral dysbiosis and its potential impact on firefighters’ health.

## 5. Conclusions

A significant shift in oral microbiome (oral dysbiosis) was observed in firefighters, both in terms of microbial proportion and diversity. An overall decrease in Firmicutes:Bacteroidetes ratio implied a proinflammatory oral microenvironment in firefighters. Particularly, the microbial changes were characterized by an increased proportion of the Streptococcaceae family (*Streptococcus* was the most dominant member) and decreased proportions of several bacterial families, including Campylobacteraceae, Enterobacteriaceae, Shewanellaceae, Pseudomonadaceae, and Fusobacteriaceae, in firefighters. Genus/species-level analysis revealed substantive qualitative and quantitative (+35.87-fold to −26,370.5-fold) changes involving several species, the majority of which showed a decreased abundance, particularly *Pseudomonas* (−26,370.5-fold), whereas a few others, particularly *Moraxella* (+35.87-fold), showed an increased abundance. Interestingly, some genera/species, such as *Uruburuella*, *Oceanotoga*, *Rhodococcus*, *Nitrincola*, and *Burkholderia* sp., were detected exclusively in the firefighters. Consumption of PAH-containing foods and tobacco use were identified as specific modifiers of oral microbial diversity in the firefighter group. Because some of these changes in microbiome have been associated with cancers and other systemic diseases in human patient populations, the observed shift in oral microbiome in the current study may imply its potential role in predisposing firefighters to occupational health risks and diseases. Further studies with a larger sample size, in conjunction with controlled experimental studies using animal models, could give additional insight into the crosstalk between altered oral microbiome and levels of occupational exposure, stress, lifestyle factors, and its role in predisposition to associated health risks in firefighters. Such studies will also help validate the identified most impacted species as biomarkers of predisposition to occupational health risks, thereby facilitating timely intervention and/or prevention of more severe health conditions and diseases in firefighters.

## Figures and Tables

**Table 1:** Microbial families showing alterations (in terms of counts and proportion) in response to various occupational exposure and lifestyle factors in firefighters ^¶^.

Metadata Variables	Microbiome Family	Counts Changed (*p*-Value)	Proportion Changed (*p*-Value)
Firefighting experience (>5 years)	Gammaproteobacteria_unclassified	↑ 0.08	↑ 0.08
	Carnobacteriaceae	↓ 0.04	↓ 0.04
	Peptococcaceae_1	↑ 0.07	↑ 0.08
Fire events (>20)	Comamonadaceae	↑ 0.018	↑ 0.018
≥1 Number of fires of one hour or more duration within 3 months	Actinomycetaceae	↓ 0.09	↓ 0.05
	Carnobacteriaceae	↑ 0.05	↑ 0.09
Food PAH intake (within past 8 h)	Spirochaetaceae	↑ 0.001	↑ 0.005
	Lactobacillaceae	↑ 0.05	↑ 0.03
	Peptostreptococcaceae	↑ 0.036	↑ 0.033
Tobacco usage (within past 8 h)	Fusobacteriaceae	↓ 0.02	↓ 0.04
	Comamonadaceae	↓ 0.04	↓ 0.04
	Eubacteriaceae	↑ 0.045	↑ 0.029
≥3 Tins/week of tobacco consumption	Spirochaetaceae	↓ 0.02	↓ 0.01
	Peptostreptococcaceae	↑ 0.09	↑ 0.06
Mouthwash (≥1 mouthwash daily)	Fusobacteriaceae	↓ 0.051	↓ 0.051
	Comamonadaceae	↓ 0.041	↓ 0.041
	Spirochaetaceae	↓ 0.044	↓ 0.022
Between Firefighters and Control	Bacteroidaceae	↓ 0.0002	↓ 0.0001
	Fusobacteriaceae	↓ 0.2	↓ 0.0001
	Campylobacteraceae	↓ 0.004	↓ 0.003
	Enterobacteriaceae	↓ 0.0003	↓ 0.0003
	Shewanellaceae	↓ 0.0005	↓ 0.0005
	Pseudomonadaceae	↓ 0.0018	↓ 0.0019
	Staphylococcaceae	↓ 0.086	↓ 0.093
	Bacilli_unclassified	↑ 0.002	↑ 0.011
	Aerococcaceae	↑ 0.064	↑ 0.1
	Streptococcaceae	↑ 0.0002	↑ 0.014
	Clostridiales_Incertae_Sedis_XI	↓ 0.051	↓ 0.021
	Clostridiales_unclassified	↓ 0.068	↓ 0.072
	Peptococcaceae_1	↓ 0.014	↓ 0.008
	Caulobacteraceae	↓ 0.037	↓ 0.037
	Mycobacteriaceae	↓ 0.0007	↓ 1
	Nocardiaceae	↑ 0.003	↑ 0.003
	Thermotogales_incertae_sedis	↑ 0.031	↑ 0.037
	Oceanospirillaceae	↑ 0.079	↑ 0.079
	Burkholderiaceae	↑ 0.0004	↑ 0.004
	Rhizobiaceae	↓ 0.07	↓ 0.0004

^¶^ The arrows represent an increase (upward arrow) or decrease (downward arrow) in the bacterial counts or proportion. *p*-value ≤ 0.05 showed significant alteration.

**Table 2:** Most impacted bacterial genera (>2-fold) in the oral microbiota of firefighters.

Family	Genus	Fold Change *	OTU Count (FF/Control)	Family	Genus	Fold Change *	OTU Count (FF/Control)
Pseudomonadaceae	*Pseudomonadaceae_unclassified*	−1124.5	2/2249	Eubacteriaceae	*Eubacterium*	−3.54	7.34/2597
	*Pseudomonas*	−26,370.5	2/5274	Bacteroidales_incertae_sedis	*Phocaeicola*	−2.75	12/33
*Bacteroidales_unclassified*	*Bacteroidales_unclassified*	+2.56	34,201/13,308	Cardiobacteriaceae	*Cardiobacterium*	−2.36	184/435
Fusobacteriaceae	*Fusobacterium*	−2.33	24,860/57,774	Leptotrichiaceae	*Leptotrichiaceae_unclassified*	+2.06	3442/1665
Burkholderiales_unclassified	*Burkholderiales_unclassified*	−2.97	576/1711	Campylobacteraceae	*Arcobacter*	−13	1/13
					*Campylobacter*	−7.03	1263/8883
Neisseriaceae	*Bergeriella*	−4.66	3/14		*Sulfurospirillum*	---	0/1
	*Conchiformibius*	+7.5	15/2	Shewanellaceae	*Shewanella*	−25	2/50
	*Eikenella*	−2.5	2/5				
	*Neisseri* *a*	−3.98	141,957/564,929				
	*Uruburuella*	+++	3/0				
Moraxellaceae	*Moraxella*	+35.87	287/8	Gammaproteobacteria_unclassified	*Gammaproteobacteria_unclassified*	−14.04	44/618
	*Enhydrobacter*	---	0/3				
	*Moraxellaceae_unclassified*	+4.6	23/5				
	*Psychrobacter*	---	0/3				
Spirochaetaceae	*Treponema*	−10.14	495/5021	Mycoplasmataceae	*Mycoplasma*	−7.33	77/565
	*Spirochaetaceae_unclassified*	---	0/2		*Ureaplasma*	---	0/3
Bacteroidaceae	*Bacteroides*	−4.49	85/382	Thermotogales_incertae_sedis	*Oceanotoga*	+++	3/0
SR1_family_incertae_sedis	*SR1_genus_incertae_sedis*	−9.26	771/7141	Staphylococcaceae	*Staphylococcus*	−3.93	15/59
					*Jeotgalicoccus*	---	0/1
Enterococcaceae	*Enterococcaceae_unclassified*	+2.67	24/9	Bacilli_unclassified	*Bacilli_unclassified*	+2.09	4954/2369
	*Enterococcus*	+++	2/0				
Lactobacillaceae	*Lactobacillaceae_unclassified*	−7.9	10/79	Aerococcaceae	*Abiotrophia*	+2.39	8682/3626
	*Lactobacillus*	−13.93	113/1574		*Aerococcaceae_unclassified*	+2.87	23/8
Clostridiales_Incertae_Sedis_XI	*Anaerococcus*	---	0/4	Clostridiales_Incertae_Sedis_XIII	*Anaerovorax*	−53	1/53
	*Clostridiales_In certae_Sedis_XI_unclassified*	−2.25	4/9				
	*Parvimonas*	−5.48	517/2836		*Clostridiales_Incertae_Sedis_XIII_unclassified*	−19	3/57
	*Helcococcus*	---	0/1				
	*Peptoniphilus*	+4	4/1				
Peptococcaceae_1	*Peptococcus*	−13.26	15/199		*Mogibacterium*	−2.22	698/1548
	*Peptococcaceae_1_unclassified*	---	0/1				
Peptostreptococcaceae	*Acetoanaerobium*	+++	1/0	Clostridiales_unclassified	*Clostridiales_unclassified*	−2.49	991/2473
	*Filifactor*	−10.18	220/2244				
	*Peptostreptococcaceae_unclassified*	−7.83	30/235				
Enterobacteriaceae	*Enterobacteriaceae_unclassified*	−585	13/7605	Mycobacteriaceae	*Mycobacterium*	---	0/7
	*Escherichia_Shigella*	---	0/1	Nocardiaceae	*Rhodococcus*	+++	13/0
	*Salmonella*	---	0/1	Oceanospirillaceae	*Nitrincola*	+++	3/0
	*Serratia*	---	0/5		*Oceanospirillaceae_unclassified*	+++	1/0
	*Yersinia*	---	0/25	Burkholderiaceae	*Burkholderia*	+++	30/0
Caulobacteraceae	*Brevundimonas*	---	0/11		*Burkholderiaceae_unclassified*	+++	1/0
Rhizobiaceae	*Rhizobium*	---	0/5		*Ralstonia*	+++	4/0

* Fold change: ‘−’ value represents a fold-decrease in the firefighter group compared to the control group; ‘+’ value represents a fold-increase in the firefighter group compared to the control group. ‘+++’ Depicts the bacterial species differentially detectable in the firefighter group (but not in the control group). ‘---’ Depicts the bacterial species differentially detectable in the control group (but not in the firefighter group). Abbreviations: FF = Firefighters; OTU = Operational Taxonomical Units.

## Data Availability

The original contributions presented in this study are included in the article/Supplementary Materials. Further inquiries can be directed to the corresponding author.

## Supplementary Materials

The following supporting information can be downloaded at: https://www.mdpi.com/article/10.3390/microorganisms13051154/s1. Figure S1. Oral microbiota families impacted > 2-fold in firefighters as compared to the control group. The relative impact in terms of fold-change was calculated based on means of the OTU counts in firefighter group versus the control group.
