# Supplementary material for: Oral Microbiota Dysbiosis in Firefighters and the Potential Contributing Environmental and Lifestyle Factors Based on a Case-Control Study
Source: Microorganisms. 2025 May 18;13(5):1154. doi: 10.3390/microorganisms13051154 (PMC12114565; doi:10.3390/microorganisms13051154)
Supplement: Supplementary file 1 [file microorganisms-13-01154-s001.zip › microorganisms-3588162-supplementary.pdf]

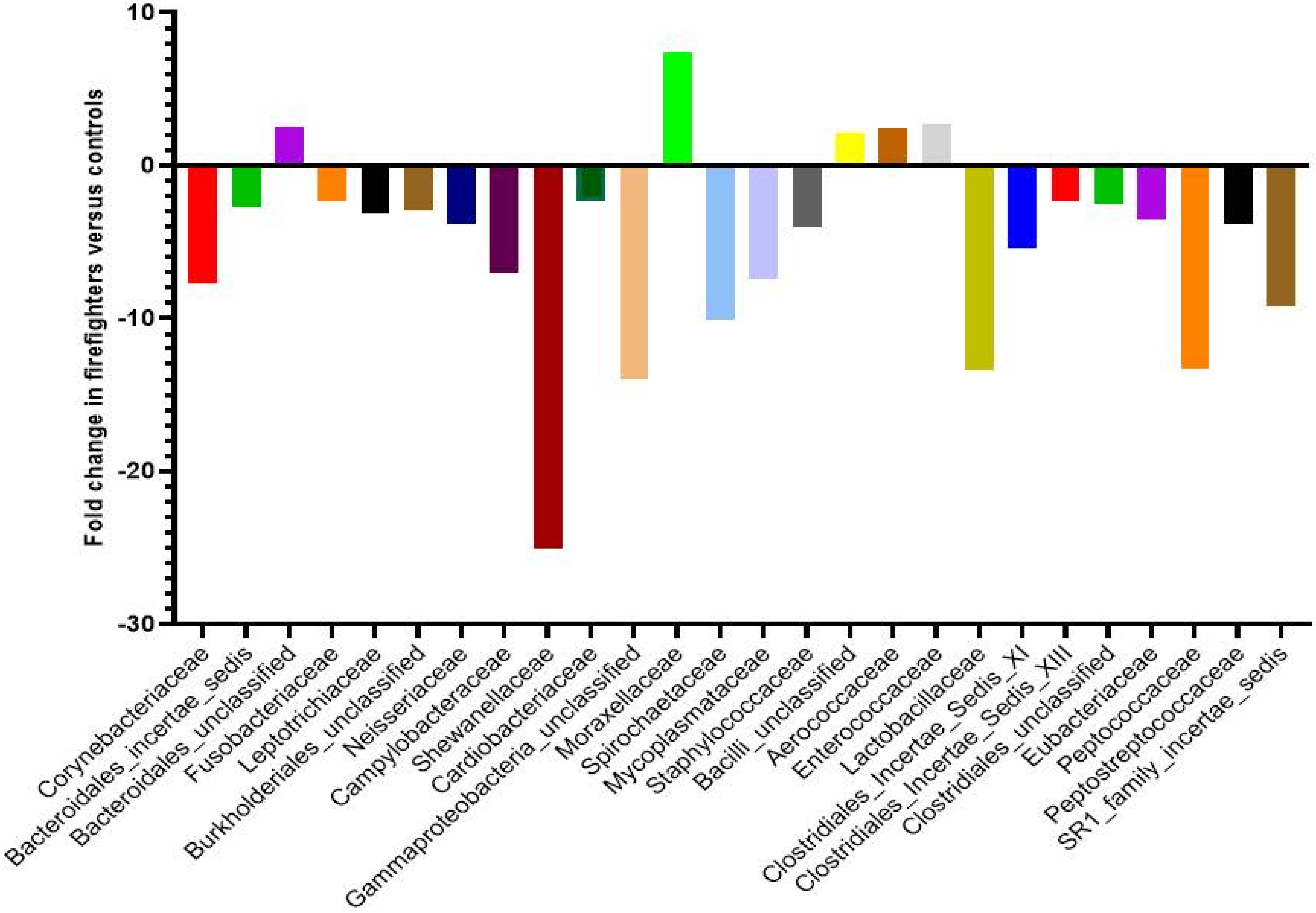

Supplementary Figure 1. Oral microbiota families impacted ≥ 2-fold in firefighters as compared to the control group. The relative impact in terms of fold-change was calculated based on means of the OTU counts in firefighter group versus the control group.
